# Supplementary material for: The primary mechanism for highly potent inhibition of HIV-1 maturation by lenacapavir
Source: PLoS Pathog. 2025 Jan 27;21(1):e1012862. doi: 10.1371/journal.ppat.1012862 (PMC11892807; doi:10.1371/journal.ppat.1012862)
Supplement: S1 Table — (DOCX) [file ppat.1012862.s001.docx]

**S1 Table. Production of HIV-1 particles.**

| **pNL4.3, µg** | **p24 ng/ml** |
| --- | --- |
| **0.03125** | **14.2 ± 0.5** |
| **0.125** | **153.6 ± 6.5** |
| **0.5** | **3504.3 ± 83.8** |
| **2.0** | **4690.5 ± 647.4** |
