# Supplementary material for: The primary mechanism for highly potent inhibition of HIV-1 maturation by lenacapavir
Source: PLoS Pathog. 2025 Jan 27;21(1):e1012862. doi: 10.1371/journal.ppat.1012862 (PMC11892807; doi:10.1371/journal.ppat.1012862)
Supplement: S2 Table — (DOCX) [file ppat.1012862.s002.docx]

**S2 Table. Data collection and refinement statistics.**

|  | NTD_LEN_ (PDB ID: 8V23) |
| --- | --- |
| **Wavelength** | 1.072156 |
| **Resolution range** | 45.8 - 2.0 (2.072 - 2.0) |
| **Space group** | P 2_1_ 2_1_ 2_1_ |
| **Unit cell** | 32.218 44.127 91.608 90 90 90 |
| **Total reflections** | 57809 (5804) |
| **Unique reflections** | 9274 (896) |
| **Multiplicity** | 6.2 (6.5) |
| **Completeness (%)** | 99.37 (100.00) |
| **Mean I/sigma(I)** | 18.82 (8.99) |
| **Wilson B-factor** | 25.61 |
| **R-pim** | 0.03553 (0.09962) |
| **CC1/2** | 0.996 (0.986) |
| **CC*** | 0.999 (0.996) |
| **Reflections used in refinement** | 9267 (896) |
| **Reflections used for R-free** | 975 (103) |
| **R-work** | 0.2301 (0.2524) |
| **R-free** | 0.2859 (0.2973) |
| **CC(work)** | 0.891 (0.637) |
| **CC(free)** | 0.898 (0.617) |
| **Number of non-hydrogen atoms** | 1187 |
| **macromolecules** | 1133 |
| **ligands** | 0 |
| **solvent** | 54 |
| **Protein residues** | 146 |
| **RMS(bonds)** | 0.009 |
| **RMS(angles)** | 1.05 |
| **Ramachandran favored (%)** | 96.53 |
| **Ramachandran allowed (%)** | 1.39 |
| **Ramachandran outliers (%)** | 2.08 |
| **Rotamer outliers (%)** | 1.64 |
| **Clashscore** | 3.98 |
| **Average B-factor** | 39.86 |
| **macromolecules** | 39.90 |
| **solvent** | 39.01 |

Statistics for the highest-resolution shell are shown in parentheses.
