# Supplementary figures and images for: The primary mechanism for highly potent inhibition of HIV-1 maturation by lenacapavir
Source: PLoS Pathog. 2025 Jan 27;21(1):e1012862. doi: 10.1371/journal.ppat.1012862 (PMC11892807; doi:10.1371/journal.ppat.1012862)

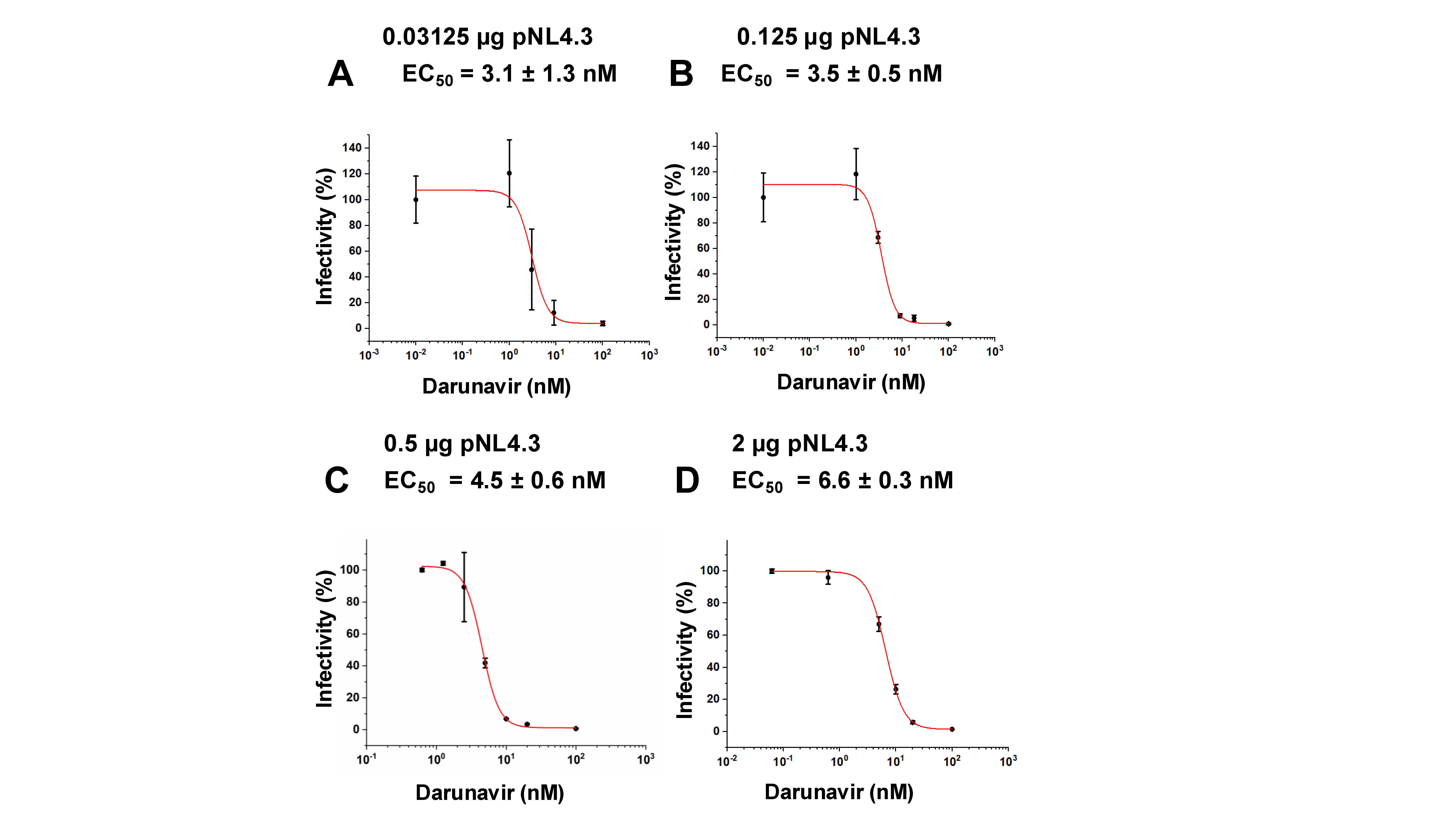

Supplement: S1 Fig — HIV-1 virions were produced by transfecting indicated amounts of the full-length, WT HIV-1NL4.3 plasmid in HEK293T cells (producer cells). Indicated concentrations of DRV or DMSO control were added to HEK293T cells, the excess DRV was removed by the Lenti-X concentrator, and the virions were used to infect HeLa TZM-bl cells (Target cells). After 48 h of infection, luciferase activity was measured to determine the EC50 values for DRV. The averaged data (+/−SD) from three independent experiments are shown. (TIFF) [file ppat.1012862.s003.tiff]

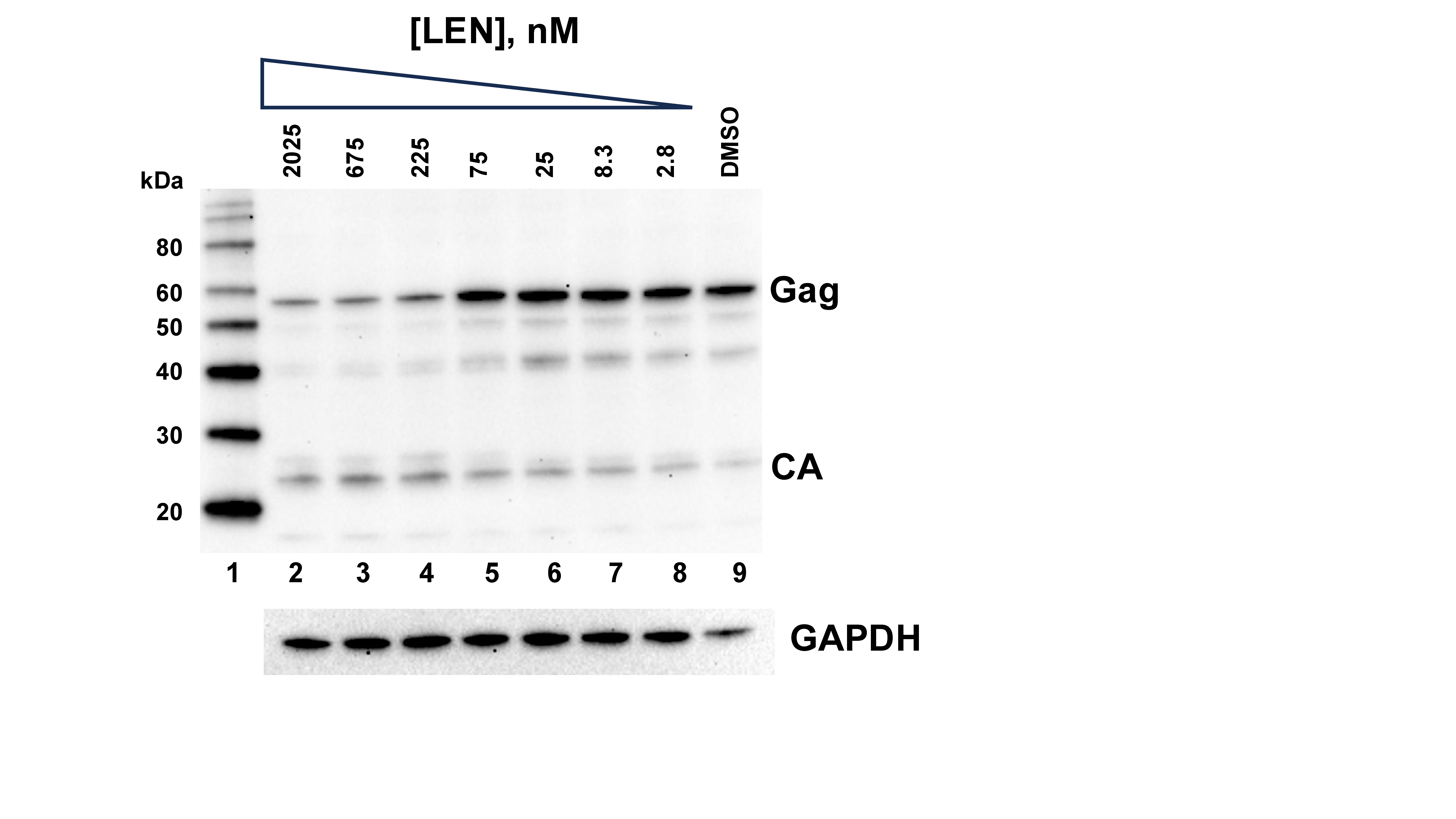

Supplement: S2 Fig — HEK293T cells were transfected with 2 µg full-length, WT HIV-1NL4.3 plasmid. After 4 h, increasing concentrations of LEN were added to the virus producer cells. After 48 h, cells were collected, and immunoblotting analysis was performed using anti-HIV1 p55 + p24 + p17 antibody (ab63917). GAPDH was used for internal control. Lane 1: molecular weight markers; lane 2: 2025 nM LEN; lane 3: 675 nM LEN; lane 4: 225 nM LEN; lane 5: 75 nM LEN; lane 6: 25 nM LEN; lane 7: 8.3 nM LEN; lane 8: 2.8 nM LEN; lane 9: DMSO control. The immunoblot is representative of results observed in two independent experiments. (TIFF) [file ppat.1012862.s004.tiff]

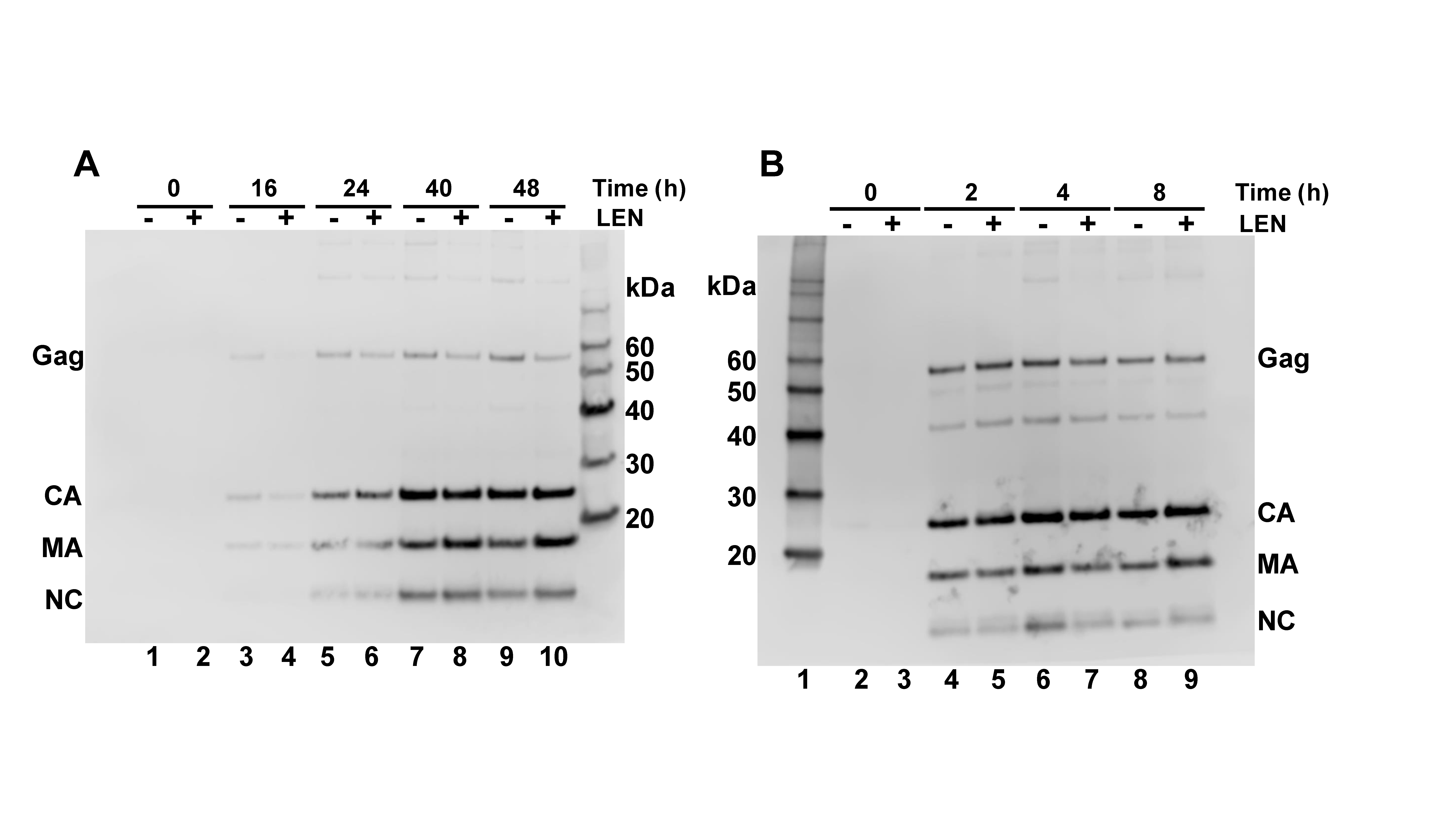

Supplement: S3 Fig — (A) HEK293T cells were transfected with 0.125 μg full-length WT HIV-1 NL4.3 plasmid. After 6 h post transfection, the medium was removed and 1 nM LEN or DMSO control containing media were added to the virus producer cells. The supernatants containing viruses were collected at 0 h, 16 h, 24 h, 40 h, 48 h, ultracentrifuged, and analyzed by immunoblotting. (B) HEK293T cells were transfected with 2 μg full-length WT HIV-1 NL4.3 plasmid. After 30 h post transfection, the medium was removed and 50 nM LEN or DMSO containing media were added to the virus producer cells. The supernatants containing virions were collected at 0 h, 2 h, 4 h, 8 h, ultracentrifuged, and analyzed by immunoblotting. Representative images of at least three independent experiments are shown in A and B. The observed Gag proteolytic processing products including CA, matrix (MA) and nucleocapsid (NC) are indicated. (TIFF) [file ppat.1012862.s005.tiff]

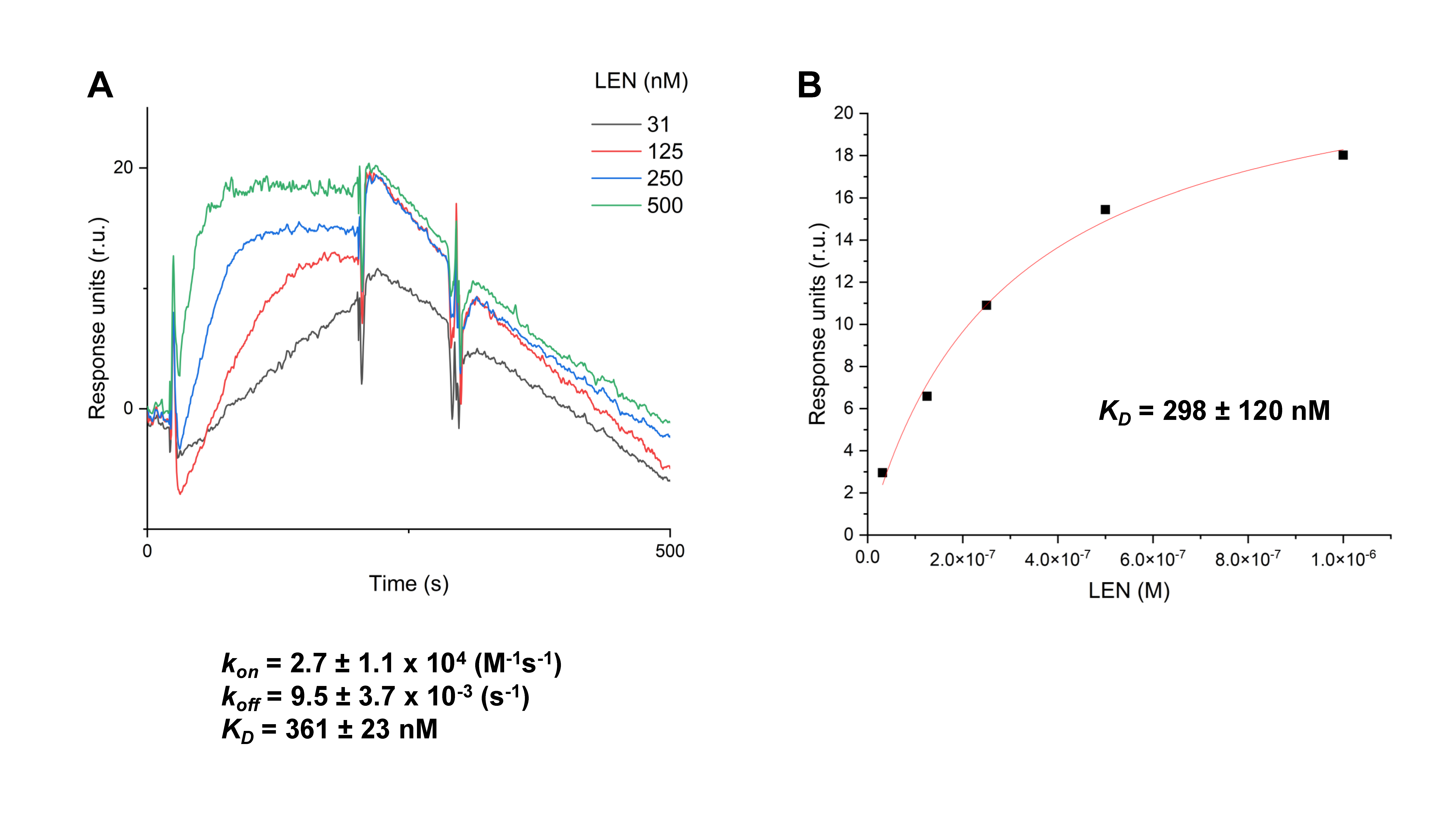

Supplement: S4 Fig — (A) Representative SPR sensorgrams of LEN binding to full-length Gag showing association (crescent curve) and dissociation (decrescent curve); KD, kon and koff values are indicated. (B) The KD value is determined using the Hill fit for (A). (TIFF) [file ppat.1012862.s006.tiff]

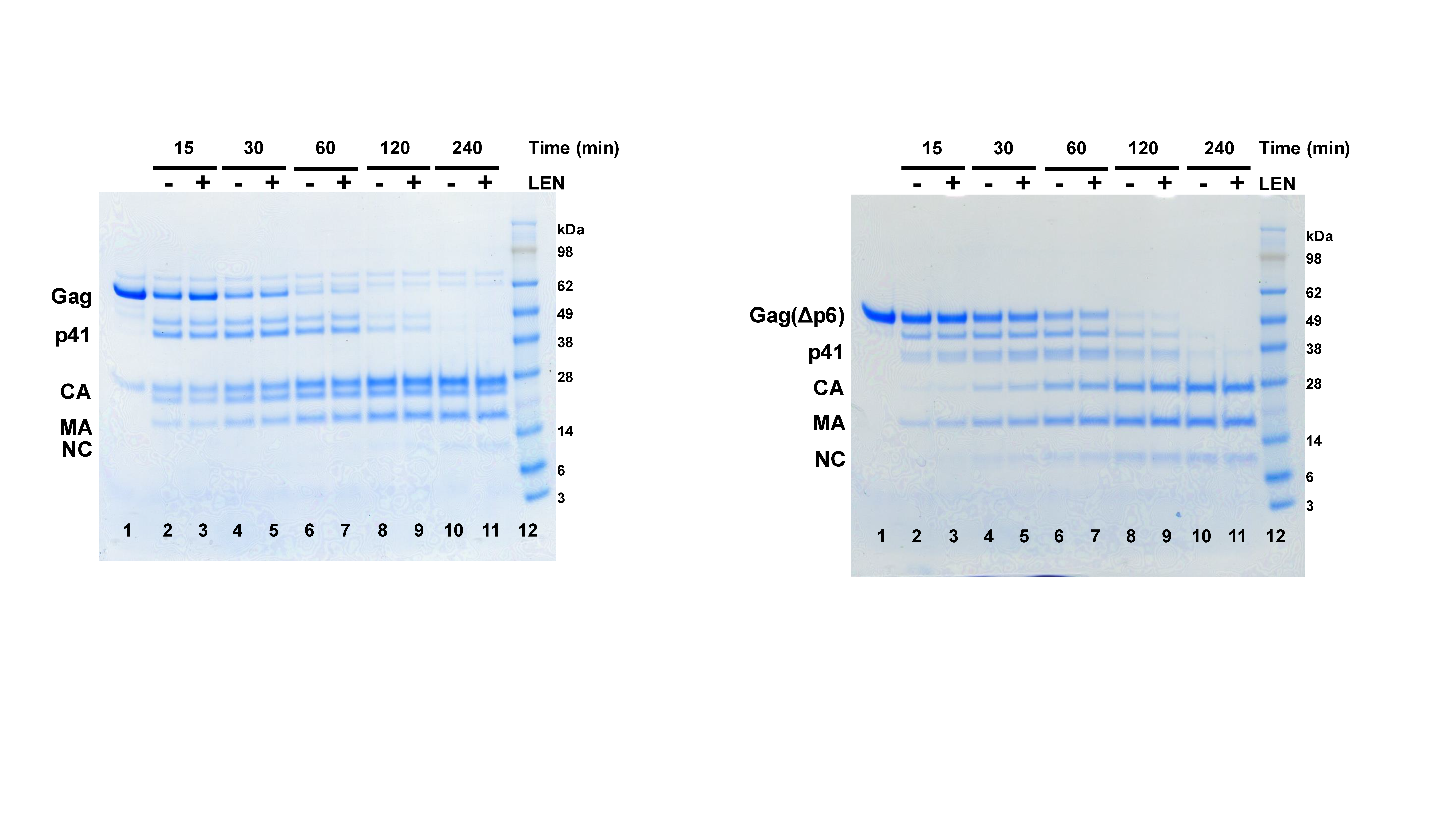

Supplement: S5 Fig — (A) HIV-1 protease mediated cleavage of full-length, recombinant Gag (1 µM) was performed in the presence of 2 µM LEN (+) or DMSO control (−). Lane 1: input of full-length Gag. Lanes 2–11: reaction products. Lane 12: MW markers. (B) HIV-1 protease mediated cleavage of 1 µM Gag(Δp6) was performed in the presence of 2 µM LEN (+) or DMSO control (−). Lane 1: input of Gag(Δp6). Lanes 2–11: reaction products. Lane 12: MW markers. Representative SDS-PAGE images of at least three independent reactions are shown. The proteolytic products including the bands corresponding to p41, CA, MA and NC are indicated. (TIFF) [file ppat.1012862.s007.tiff]

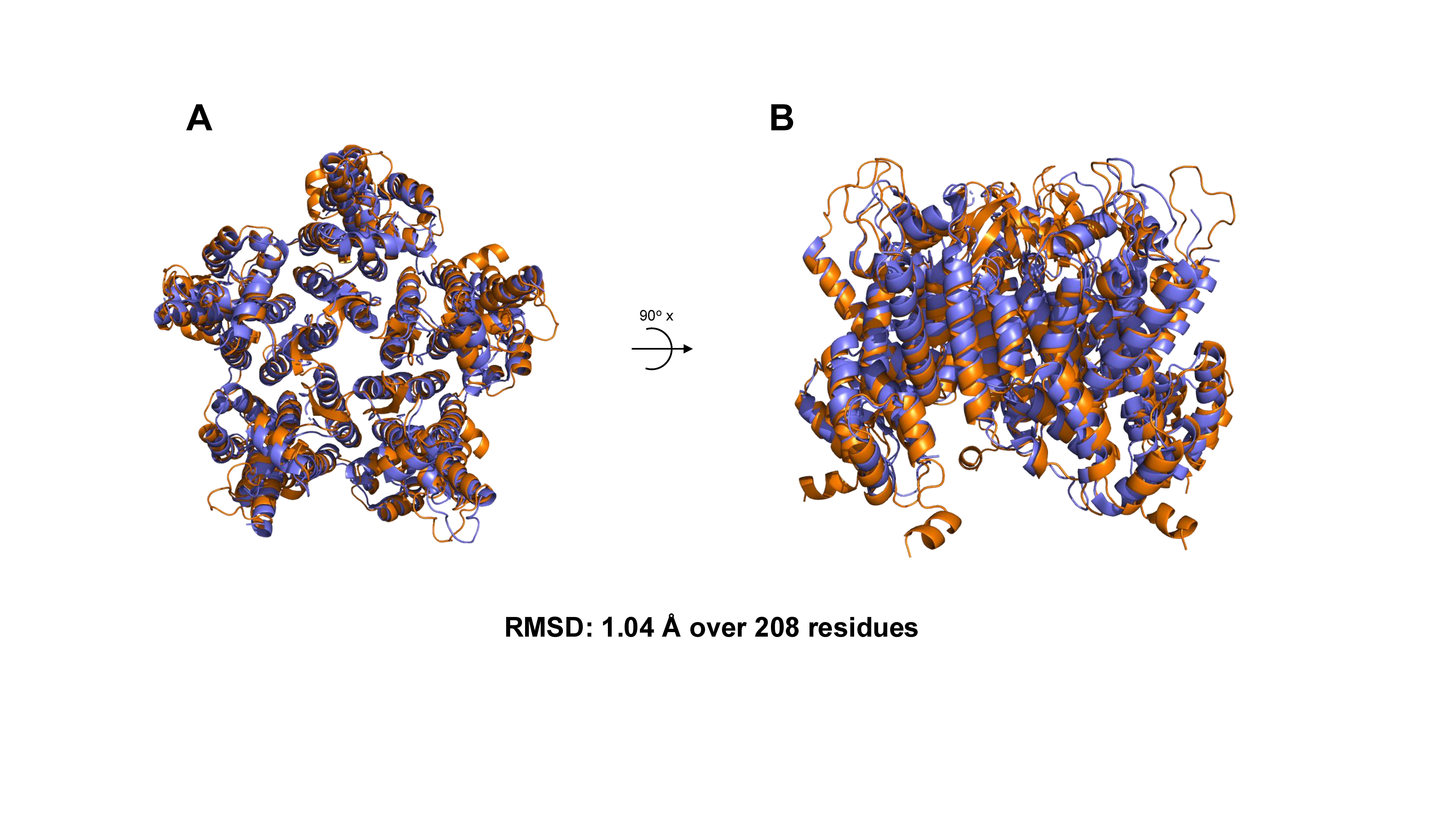

Supplement: S6 Fig — Superimposition of the AlphaFold2 generated CA pentamer structure (orange) and X-ray crystallographic structure of the cross-linked pentamer (PDB id: 3PO5, in purple); (A) top and (B) side views. (TIFF) [file ppat.1012862.s008.tiff]

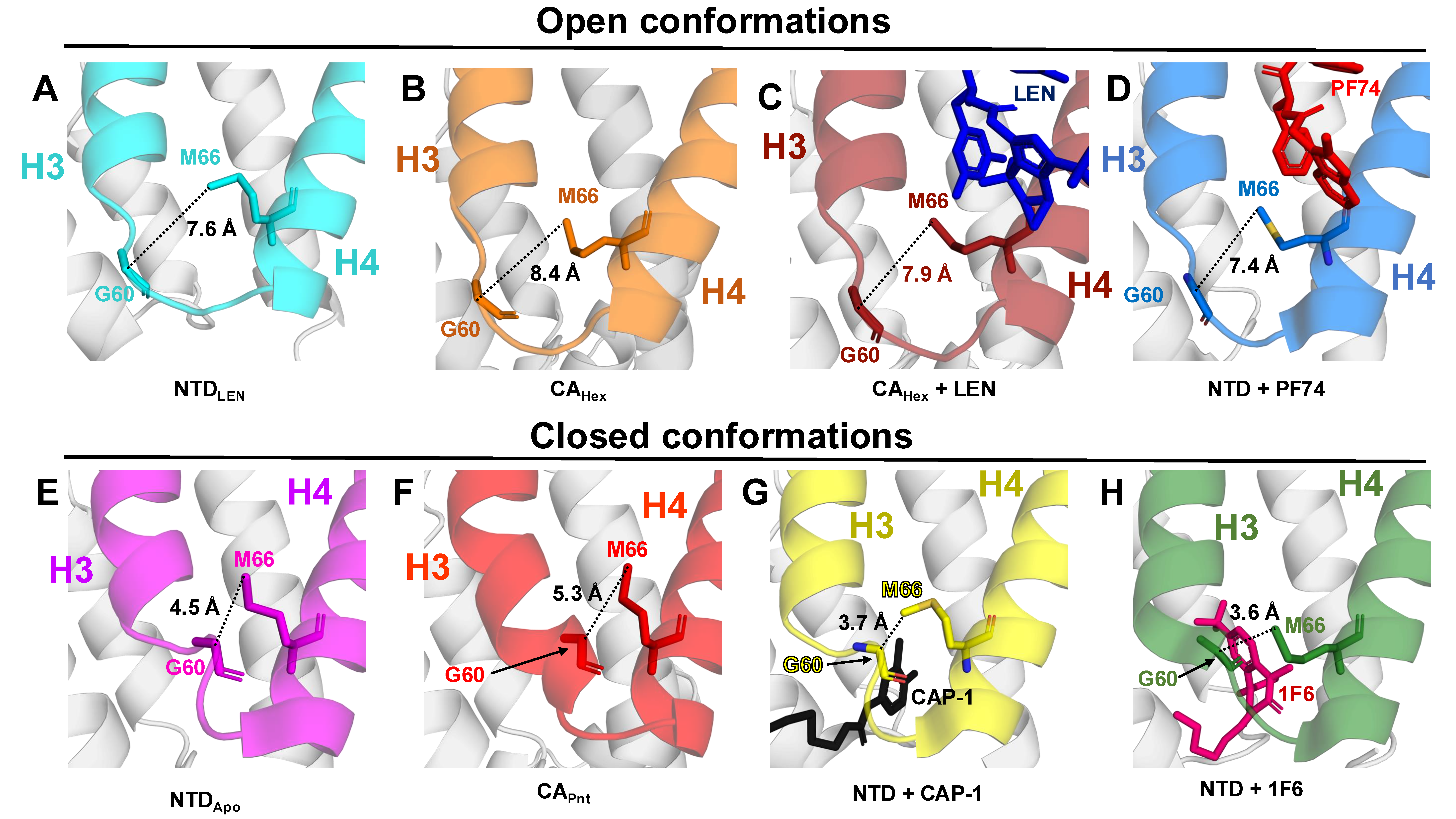

Supplement: S7 Fig — The distances between Cα of G60 and Cε of M66 are shown to delineate the opened and closed conformations. (A) NTDLEN (PDB: 8V23, present work); (B) CAHex (PDB: 7URN); (C) CAHex + LEN (PDB 6VKV); (D) NTD + PF74 (PDB: 2XDE); (E) NTDApo (PDB: 5HGK); (F) CAPnt (PDB: 7URN); (G) NTD + CAP-1 (PDB: 2JPR); (H) NTD + 1F6 (PDB: 4INB). Colored protein segments indicate connecting helices H3 and H4. Black dash lines indicate distances between Cα of Gly60 and Cε of Met66. (TIFF) [file ppat.1012862.s009.tiff]

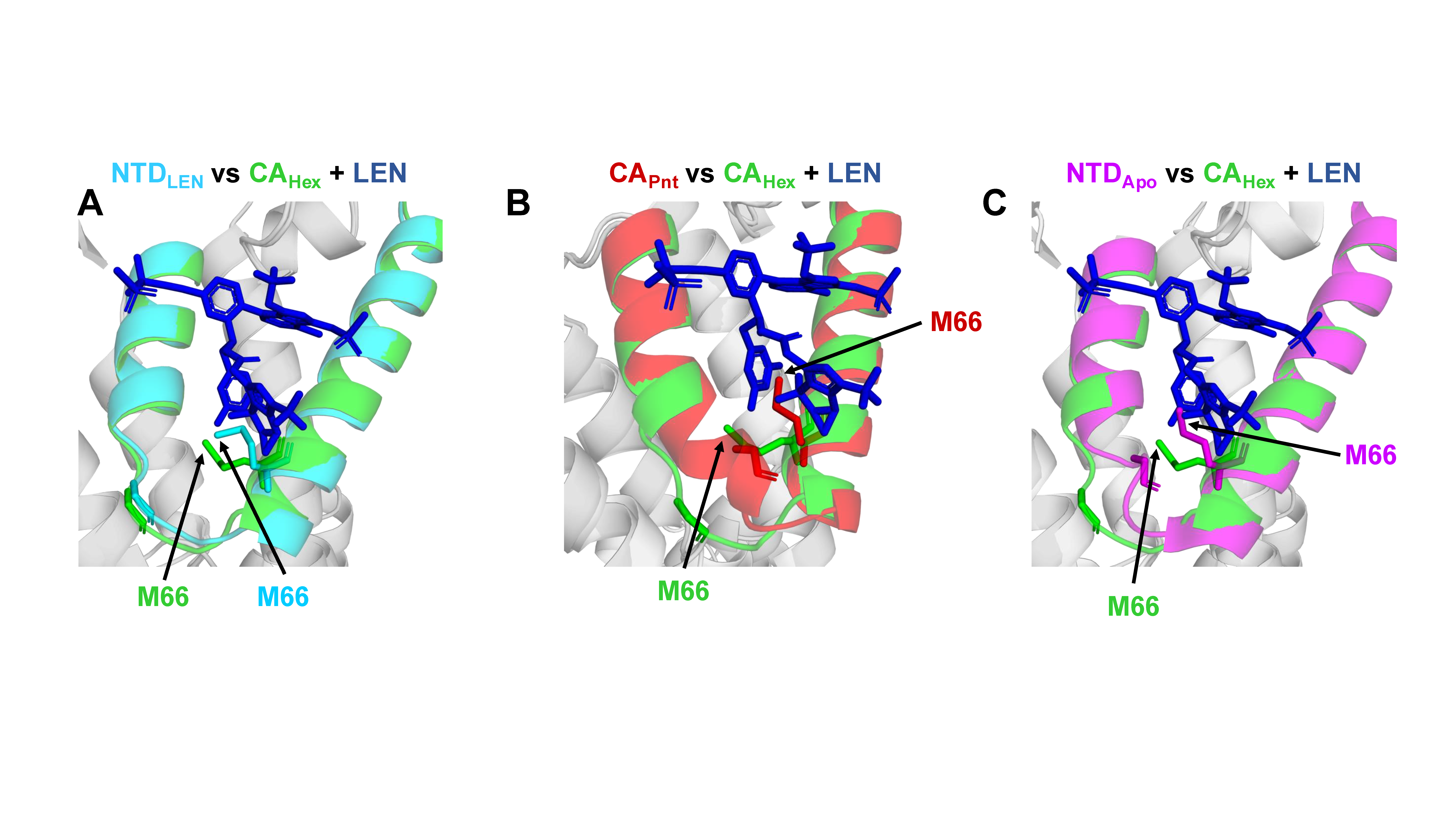

Supplement: S8 Fig — Structure of CAHex (Green) + LEN (dark blue) (PDB: 6VKV) superimposed onto (A) NTD + LEN (NTDLEN, steel blue, PDB: 8V23, present work); (B) CAPnt (scarlet, PDB: 7URN); and (C) NTDApo (magenta, PDB: 5HGK). Different conformations of Met66 side chains are shown. In panel A, the Met66 side chain is compatible with LEN binding to the opened conformation seen in NTDLEN. In contrast, the Met66 side chains in native CAPnt (B) and NTDApo (C) encounters steric hindrance with respect to LEN. (TIFF) [file ppat.1012862.s010.tiff]
