# Supplementary material for: The primary mechanism for highly potent inhibition of HIV-1 maturation by lenacapavir
Source: PLoS Pathog. 2025 Jan 27;21(1):e1012862. doi: 10.1371/journal.ppat.1012862 (PMC11892807; doi:10.1371/journal.ppat.1012862)
Supplement: Attachment — Submitted filename: Rebuttal_Rev2.pdf [file ppat.1012862.s012.pdf]

Point-by-point responses to Editor’s comments

Q. The mass photometry in Figure 6 is impressive. However, the text describes data in the presence of LEN after 1 hour, but 10 min and 16h are shown? Assuming all panels are accurately labelled, since the data in D/E show 10 min/1hr in presence of IP6, it seems useful to compare both conditions at 1 hr and 16hr. Based on the text, one might infer this has been done, so I would consider this an editorial revision.
A. We have included these results in revised Fig 6 and updated the respective Figure legend (page 40) and the Results section (page 13).

Q. Please describe in the text how you evaluated p24 levels, measured by ELISA, to infer the number of CA molecules and in turn, what this means about the molecules of LEN that can affect virion infectivity, given that a substantial amount of CA is not assembled into the core during viral maturation. This will both clarify the utility of this analysis to readers outside of our field but also provide interesting context for readers more familiar with the topic.
A. See the added text in the Results section at the bottom of page 10 and the beginning of page 11. Furthermore, we have included additional experimental details in Materials and Methods (see page 24).

Q. Please reconsider the use of “excessively high concentrations” in the discussion describing reference 35. Acknowledging that it is much higher than physiological concentrations seems quite appropriate, but “excessively high” could be viewed as unnecessary editorializing.
A. We have revised the statement as follows (see page 19): “However, this structural study used a concentration of inhibitor (300 μM) that greatly exceeded (>100,000-fold) what is required to inhibit HIV-1 replication under physiological conditions (37)”.

Q. Despite it being a supplementary figure, S2 would really be much nicer and accessible if the LEN concentrations were included in the actual figure rather than the legend.
A. We have revised S2 Fig accordingly.

Q. Technical Check requests. We note that you have included "Author Summary" in the manuscript file. Please note that THIS SHOULD BE 150-200 WORDS LONG.
A. We have extended our Author Summary to meet the above requirement.

This concludes our rebuttal.
