# Supplementary material for: The primary mechanism for highly potent inhibition of HIV-1 maturation by lenacapavir
Source: PLoS Pathog. 2025 Jan 27;21(1):e1012862. doi: 10.1371/journal.ppat.1012862 (PMC11892807; doi:10.1371/journal.ppat.1012862)
Supplement: Attachment — Submitted filename: Rebuttal_Final.docx [file ppat.1012862.s011.docx]

PLoS Pathogens

Dear Editor,

Thank you very much for your erudite review of our manuscript. We particularly appreciate your taking time to attentively read our manuscript and provide your thoughtful feedback, which we found very helpful. We thank Reviewer 1 for positive remarks. Reviewer 3 has been incredibly helpful by suggesting replacing Lenti-X with 20% sucrose cushion. We had to redo the LC-MS/MS experiments, which was significant undertaking, but the quality of new results in revised Fig 3 justifies the effort. We always appreciate thoughtful and constructive criticism! Reviewer 2 seemed to misinterpret some of our data (S2 Fig) and made suggestions, which are refuted by the new data presented in the revised manuscript. A surprisingly assertive tone of Reviewer 2’s remarks might have been affected by the recent structural study on LEN interactions with VLPs, which was conducted under pharmacologically irrelevant, excessively high (300 µM) concentrations of LEN. Furthermore, no corroborating mechanistic results were presented in the recently published preprint (see Discussion in the revised manuscript and the rebuttal below).

The key new findings that substantially strengthened our original story include:

1. We now present highly innovative and exciting mass photometry data in Fig 6, which for the first time reports on assembly intermediates of WT CA + IP6 vs WT CA + LEN. Specifically, Fig 6 shows striking differences between these two reactions and reveals new clues that LEN strongly stabilizes CA dimers thereby preventing formation WT CA pentamers, which require an odd number of closed CA subunits.
2. We included new virology and biochemistry data (S3-S5 Figs) for LEN interactions with Gag, which show that LEN does not impar proteolytic processing of Gag in virions or *in vitro*.
3. New Fig 9 was generated in collaboration with Dr. Francis enabling us to demonstrate that LEN hyper-stabilized both WT and M66A cores, albeit substantially higher concentrations of the inhibitor were needed to elicit these effects on the mutant vs. WT virus suggesting that the M66A substitution confers substantial resistance to LEN.

Collectively, because of constructive comments from you and all reviewers, we conducted substantial additional research which provided further insights into highly potent antiviral mode of action of LEN during virion maturation. The revised text in the manuscript is in blue. Your summary comments are answered in the context of our responses to reviewers’ critique. Point-by-point responses to reviewers’ comments are below.

Sincerely,

On behalf of all authors

Mamuka Kvaratskhelia

Szu-Wei Huang

Point-by-point responses to reviewers’ comments:

**Reviewer 1.**

**Major issue:** None

**Minor Issues:**

Q. 1) Lines 265-268: The authors note that hexamer field for CA(M66A) is lower than WT, but no data is shown in Fig.5 regarding yield. Is it possible to quantify the yields?

A. We removed the above statement and instead, present new data with HIV-1 (M66A) cores in main Fig 9, which provides more accurate estimation of the resistance levels of the M66A cores to LEN.

Q. 2) S1 Fig. – The figure legend states LEN was removed; this appears to be an error since this figure shows DRV treatment.

A. Thanks for spotting the typo. It is now corrected.

Q. 3) S3 Fig. - Panel and axis labels are missing for B and C.

A. They were lost during the pdf conversion. We’ll doublecheck this time that the figure is converted as intended.

Q. 4) I suggest moving S3 and (especially) S4 Fig to the main text, perhaps combining with Fig. 3.

A. Agreed. See new main Figs 2 and 4.

Q. 5) The model (S7 Fig) should also be included in the main body of the report.

Agreed. See new Fig 10.

**Reviewer #2:**

**Q.** (summary) …the interpretation seems overdone. Maturation is a kinetic process that involves cleavage of sites in Gag and Gag-Pol at different rates, disassembly of the immature lattice and assembly of the mature lattice, and closure of the capsid via pentamer formation. How pentamers form during HIV-1 capsid assembly is unknown. Since the authors performed no kinetic analyses of the effects of Len on lattice formation, they have ignored a possible simple explanation for the apparent impairment in biochemical, structural and/or architectural maturation. More importantly, some of the results may have alternative interpretations.

A. Based on published data by Gilead and our own corroborating unpublished results it was already clear to us that LEN did not influence Gag proteolytic processing. However, we failed to clarify this in our original submission. We now include new virology and biochemistry results in S2, S3 and S5 Figs which speak for themselves. Furthermore, we are glad that a preprint on a structural study on LEN interactions with Gag in the context of VLPs has been published in bioRxiv allowing us to comment on the experimental conditions used in this study. Please read second and third paragraphs of Discussion.

**Major issues:**

Q. 1. The authors employ Cys mutants that stabilize hexameric and pentameric capsomeres by disulfide bond formation. They observed no effect on formation of crosslinked hexamers but inhibition of crosslinked pentamers. Notwithstanding the risk associated with interpreting results of Len on assembly of mutant proteins in vitro with respect to maturation of wild type HIV-1, it has been demonstrated that the structure of the pentamers formed by A21C/A22C mutant CA protein is markedly different from that of the pentamer present in native HIV-1 cores (PMID: 27980210). Thus, the relevance of the observed effect of Len on pentamer formation in this experiment may not be relevant to Len's effects on HIV-1 maturation.

A: Please consider the following:

i) We understand your reservations about using cross-linked pentamers and discuss them in the revised manuscript (see lines 241-252). At the same time, the caution should be exercised comparing isolated cross-linked pentamer with the pentamer in the capsid that tightly interacts with five surrounding hexamers. Here, we are studying capsid assembly intermediates, and the fact is that the structure of isolated WT CA pentamer is not known. Therefore, we used AlphaFold to build the model for the isolated WT CA pentamer, which suggested that the predicted structure is very similar to the cross-linked pentamer (see new Fig S6 and the respective Results section).

On a more general note, the reviewer 3 correctly commented that “the formation of dissulfide hexamers but not pentamers is a strong dataset for the premise of the article.” Indeed, while the findings in main Fig 5 formed the initial premise to perform extensive follow up studies described here, our conclusions are drawn based on corroborating data with WT CA (see points 2-4 below).

ii) We have now added new main Fig 6, which depicts highly innovative and exciting mass photometry data comparing assembly intermediates of WT CA + IP6 vs WT CA + LEN. In complete contrast from CA + IP6, which formed ample amounts of pentamers among other intermediates, LEN specifically formed dimers and hexamers but no pentamers. See further details in new main Fig 6 and the corresponding text in Results and Discussion.
iii) Formerly S4 Fig, currently main Fig 4, which uses WT CA, provides compelling corroborating data for the results obtained with cross-linked pentamers and hexamers. This was noticed by the editor but is left out of your remarks above.
iv) Our structural data explains as to why the opened conformation of the NTD_LEN_ is not compatible with the pentamer formation.

Taken together, we have provided multiple corroborating lines of evidence for our conclusion.

Q. 2. Fig. 2: Actual Len:CA ratios in virions may have been reduced by removal of Len during virus purification and/or by loss during extraction. Therefore, the conclusion that “sub-stoichiometric LEN:CA ratios suffice to inhibit late steps of HIV-1 replication” (line 141) may be erroneous. It remains possible that the antiviral mechanism involves stoichiometric association, since Len may preferentially bind to the assembled CA in the virion vs. the CA protein that is not part of the capsid lattice, which is thought to be approximately half of the total CA in mature virions.

A. Thanks to a very helpful suggestion by reviewer 3, our methodology has been substantially improved by replacing Lenti-X with sucrose cushion. Our calculations reveal that the LEN loss throughout our assay is very minimal if any. Nearly all (>95%) LEN added to the cell culture is tightly bound to capsid. By contrast, there is no detectable LEN in control experiments without virions (see new exciting results in revised main Fig 3). The key point is that we are adding substantially sub-stoichiometric concentrations of LEN vs CA in cell culture to start with. Therefore, we cannot possibly have stoichiometric LEN:CA interactions. In fact, main Fig 3 shows that LEN EC_50_ values are observed at LEN:CA ratios of 1:24.

Q. 3. Fig. 4: the authors used CA protein with the M66A substitution for these experiments. The Ile substitution at this position is known to confer Len resistance and a large HIV-1 fitness cost, so the relevance of the approach seems doubtful. Does M66A also confer Len resistance? If so, how do the authors explain the effect of Len?

A. The clinically relevant drug-resistant M66I substitution and the artificially designed M66A mutant exhibit very different phenotypes. Our manuscript describing “Structural and Mechanistic Bases for Resistance of the M66I Capsid Variant to Lenacapavir” is currently under review, and the respective preprint is available from bioRxiv. The M66A substitution is used here to corroborate our findings with the WT CA as we believe in providing multiple lines of evidence to support our conclusions. New results in main Fig 9 show that while M66A cores exhibit substantial resistance to LEN, they are affected by higher concentrations of LEN. Again, these new results are both complementary and consistent with the biochemical results in current main Fig 8.

Q. 4. The immunoblot shown in S2 Fig is highly suggestive of impaired cleavage in Gag. This should be fully characterized, ideally through pulse-chase assays of Gag processing in infected cells and virions. Impaired Gag cleavage could well be the basis for the observed maturation defect engendered by Len.

A. S2 Fig does not anyhow suggest the impaired cleavage of Gag. Please pay attention that S2 Fig shows intracellular Gag levels which do not inform proteolytic processing of Gag during virion maturation. We now present new complementary data for Gag proteolytic processing products in virions (see S3 Fig), which refute your suggestion that “Impaired Gag cleavage could well be the basis for the observed maturation defect engendered by Len.”

**Minor Issues:**

Q. 5. Line 150: “Gag degradation…” This has not been demonstrated. The apparent decrease in Gag accumulation in transfected cells could have another explanation, such as decreased translation, accelerated budding, or reduced solubility.

A. We replaced “Gag degradation…” with “reduction of intracellular Gag levels”. FYI, our unpublished data indicate the following: 1) when we add 100 nM LEN to HEK293T cells 30 h post transfection of pNL4.3 (after ample amounts of Gag are already detected in cells) intracellular Gag levels drop markedly within several hours; 2) the virion productions are decreased concomitantly with the decrease of intracellular Gag levels: the less Gag in cells the proportionally less virions in the supernatant; 3) we boil the entire sample in SDS. Therefore, we believe we would catch both soluble and insoluble Gag; 4) intracellular Gag levels are equally reduced by LEN for WT and D25A HIV-1 protease inactive viruses; 5) intracellular Gag levels are similarly reduced by LEN in parental vs IP6 depleted cells. This is ongoing research, which is completely separate from the present manuscript. Accordingly, we state: “The mechanism by which LEN reduces Gag levels in virus producer cells is unknown and was not further investigated here. Instead, our studies focused on the more potent antiviral activity of LEN during virion morphogenesis.” (see lines 145-147). Also, unfortunately, the recent structural study which examined interactions of LEN with VLPs do not reveal any clues about how LEN modulates cellular levels of Gag.

Q. 6. Discussion lines 281-285: the level of p24 in patient plasma seems irrelevant to the matter, as it likely has little to do with the concentration of Gag in a productively infected cell. The important comparison would be the Gag concentrations in their transfected cells vs. those in infected T cells. On that note, adding a producer cell Len titration experiment using infected T cells (even a T cell line) could increase confidence in the relevance of the data.

A. We have rephrased the first paragraph of Discussion. The simple message we are trying to coney is the following. We have established inverse correlation between the LEN potency and the p24 levels in cell culture. The viremia in patients is lower than the viruses we have in cell culture. Therefore, it is logical to suggest that in the clinical setting LEN can potently (with pM EC_50_s) inhibit HIV-1 maturation.

**Reviewer #3:**

Q. (summary). The experiments measuring the structure and effect of LEN on capsid assembly are only performed in CA monomers and do not take into consideration the effects of LEN into the Gag precursor and maturation. The authors should discuss these limitations, the caveats, and what could differ in maturation from Gag to CA to capsid lattice formation.

A. See new S2, S3 and S5 Figs, respective text and our response to reviewer 2 regrading a similar comment.

Q. (summary) The lack of experiments in primary cells that are targets to HIV-1 reduces the significance of the study.

A. Gilead has already published antiviral activities of LEN is primary cells (ref#13). As you are well aware, it would be immensely challenging to conduct detailed mechanistic studies presented here in primary cells. However, our findings indicating the inverse correlation between the LEN potency and the p24 levels and demonstrating that LEN inhibits HIV-1 maturation with 60 pM EC_50_ values are in excellent agreement with very similar EC_50_ values observed in primary cells by Gilead (ref#13).

**Major Issues**

Q. 1. The usage of Lenti-X and the LEN found in supernatants without viruses decrease the excitement of the manuscript. The 3~4x lower trace amount of LEN that is found in supernatants without viruses could still represent an action in target cells and not only in the production stage. This is important as LEN has such a potency at pM doses. A sucrose cushion would result in a cleaner approach to remove LEN from the SN (as the volume of the cushion would prevent contamination, unlike using the PEG approach were there's no solution in between the phases). As an alternative so that to not perform the whole study with that methodology, it is advisable to provide LC/MS-MS data with LEN with a retrovirus known not to bind to LEN (such as MLV or HTLV). Further, infections in TZMbl using the same concentrations of LEN that are measured by LC-MS/MS would attenuate this concern.

A. Many thanks for your very thoughtful and helpful suggestions! Indeed, we were losing substantial amounts of LEN because of Lenti-X. Carry over amounts of LEN non-specifically bound to Lenti-X in control samples were not inhibiting the target cells. Perhaps, LEN bound to PEG was not readily released in target cells, which in turn adversely affected LEN EC_50_ values and LEN:CA stoichiometry. Once we switched to 20% sucrose cushion, we could account for nearly all (>95%) of input LEN, which was specifically bound to virions as evidenced by no LEN detected in control samples. New exciting results in Figs 1 and 3 speak for themselves. Thank you once again!

Q 2: The Xray crystalography data is performed in CA monomers and the experiments with mCLPs are also performed with "pre-matured" CA mixed with LEN/IP6/others. LEN and IP6 might bind to the forming Gag precursor and this experimental model does not take into consideration the presence of LEN and IP6 at the moment of maturation. LEN binding to the Gag precursor is likely to have different interaction profiles with the different a.a. in CA.

A. We present SPR data demonstrating that LEN binds Gag with *K_D_* of >200 nM, which is 3-orders of magnitude higher than EC_50_ of 60 pM observed in our assays (S4 Fig). If your thought process is influenced by the recent structural study (ref#35), we now explain that they used 300 µM LEN, which is 5,000,000-times higher than EC_50_ of 60 pM observed in our assays. We have also added two new paragraphs to Discussion (see lines 399-413).

Q 3: From the data from Fig 1 and others, could the authors estimate how many LEN molecules per capsid would be sufficient to cause a maturation defect?

A. EC_50_ values and corresponding LEN to CA ratios are reported in Fig 3.

**Minor issues**

Q. Line 56-58 - "The integrity of the mature capsid architecture is important for protecting the viral genome from immune sensing during subsequent trafficking of HIV-1 cores throughout the cytoplasm to successfully reach the nucleus of infected target cells."

The opposite reasoning has also been shown: Only an intact capsid is targeted by TRIM5 and Mx2, which points to the opposite hypothesis. As the matter is still under debate and it's a model among many for the early steps of infection, the authors could revise the sentence.

A. Both our statement that capsid protects the viral genome from immune sensing and your valid points regarding the restriction factors TRIM5 and Mx2 recognizing the capsid lattice are true. It’s a tug of war. We have rephrased the sentence as follows: “The mature capsid is the principal interface with the cellular milieu, as it encounters both host-dependency cofactors and antiviral restriction factors during trafficking of HIV-1 cores across the cytoplasm, through the nuclear pore complex and inside the nucleus”.

Q. Line 70- The Study by the Diaz Griffero lab (PMID35005542) claims that LEN/GS-CA1 allows for nuclear import, the authors should discuss the differences and what could explain the discrepancy of the results.
A. Please note the following. The conclusion drawn in the indicated study (PMID: 35005542) is based on a single assay of fractionating the infected cells and detecting CA levels by western blotting in cytoplasmic vs nuclear fractions. Even if these assays were done extremely carefully without contaminating the nuclear fractions by cytoplasmic fractions which contain the bulk of CA, the cores that bind to the nuclear envelope but don’t make inside the nucleus would still show up in the nuclear fractions. By contrast, Gilead (ref#13) and we (ref#14) reported multiple lines of corroborating evidence that LEN blocks HIV-1 nuclear import. Since, strictly speaking, PMID: 35005542 used GS-CA1 rather than LEN and our current manuscript is not a review, we respectfully decline to reference the indicated publication.

Q. Related to Line 87 and the discussion - The authors should further discuss the biological relevance of the findings as it is unknown how many capsids are produced per cell at a given moment, in different cell types.

A. This is a good point but what ultimately matters is that viremia in patients is substantially lower (and likely in their different cell types) than virus amounts produced in our artificial HEK293K cells. Since we established inverse correlation indicating the lower p24 levels the higher is LEN potency, it is logical to suggest that LEN impairs HIV-1 maturation with pM EC_50_ values in clinical setting (see the first paragraph of discussion).

Q. Line 134/Fig2.B. The concentrations are shown in ng/mL while the rest of the manuscript shows molarity. Please convert or add the information in nM/pM for reading clarity purposes.

A. Converted. See lines: 204 and 390-394.

Q. Line 149-150. The figure showing p24 vs p55(Gag) show less Gag in the higher >75nM LEN. It is also visible a stronger p24 band. The authors should discuss this finding. The authors claim Gag "degradation", perhaps another word would be more appropriate, as no degradation studies were conducted and could be simply a difference into the protease activity efficiency.

A. See our response to reviewer 2’s similar question.

Q. Fig S3. B and C labels are absent.

A. This time we’ll doublecheck that the conversion of our figures into the pdf format goes as intended.

Q. Fig S7 model should be improved in relation with the structural findings shown in the results.

A. The model now includes our innovative finding that LEN stabilizes CA dimers.

Q. Lenacapavir source should be stated in material and methods. (and please confirm that it's lenacapavir GS-6207 and not GS-CA1).

A. Yes, our studies were conducted with LEN as clearly stated throughout the manuscript. As for the source of LEN, we have now referenced our previous publication (ref#14, see lines 476-477).

The end of the rebuttal. Thank you!
